# Chromosome-level genome assembly of a benthic associated Syngnathiformes species: the common dragonet, *Callionymus lyra*

**DOI:** 10.46471/gigabyte.6

**Published:** 2020-10-20

**Authors:** Sven Winter, Stefan Prost, Jordi de Raad, Raphael T. F. Coimbra, Magnus Wolf, Marcel Nebenführ, Annika Held, Melina Kurzawe, Ramona Papapostolou, Jade Tessien, Julian Bludau, Andreas Kelch, Sarah Gronefeld, Yannis Schöneberg, Christian Zeitz, Konstantin Zapf, David Prochotta, Maximilian Murphy, Monica M. Sheffer, Moritz Sonnewald, Maria A. Nilsson, Axel Janke

**Affiliations:** ^1^ Institute for Ecology, Evolution and Diversity, Goethe University, Frankfurt am Main, Germany; ^2^ Senckenberg Biodiversity and Climate Research Centre, Frankfurt am Main, Germany; ^3^ LOEWE-Centre for Translational Biodiversity Genomics, Frankfurt am Main, Germany; ^4^ South African National Biodiversity Institute, National Zoological Garden, Pretoria, South Africa; ^5^ Zoological Institute and Museum, University of Greifswald, Greifswald, Germany; ^6^ Senckenberg Research Institute, Department of Marine Zoology, Section Ichthyology, Frankfurt am Main, Germany

## Abstract

**Background:**

The common dragonet, *Callionymus lyra*, is one of three *Callionymus* species inhabiting the North Sea. All three species show strong sexual dimorphism. The males show strong morphological differentiation, e.g., species-specific colouration and size relations, while the females of different species have few distinguishing characters. *Callionymus* belongs to the ‘benthic associated clade’ of the order Syngnathiformes. The ‘benthic associated clade’ so far is not represented by genome data and serves as an important outgroup to understand the morphological transformation in ‘long-snouted’ syngnatiformes such as seahorses and pipefishes.

**Findings:**

Here, we present the chromosome-level genome assembly of *C. lyra*. We applied Oxford Nanopore Technologies’ long-read sequencing, short-read DNBseq, and proximity-ligation-based scaffolding to generate a high-quality genome assembly. The resulting assembly has a contig N50 of 2.2 Mbp and a scaffold N50 of 26.7 Mbp. The total assembly length is 568.7 Mbp, of which over 538 Mbp were scaffolded into 19 chromosome-length scaffolds. The identification of 94.5% complete BUSCO genes indicates high assembly completeness. Additionally, we sequenced and assembled a multi-tissue transcriptome with a total length of 255.5 Mbp that was used to aid the annotation of the genome assembly. The annotation resulted in 19,849 annotated transcripts and identified a repeat content of 27.7%.

**Conclusions:**

The chromosome-level assembly of *C. lyra* provides a high-quality reference genome for future population genomic, phylogenomic, and phylogeographic analyses.

## Data description

## Background information

**Figure 1.**
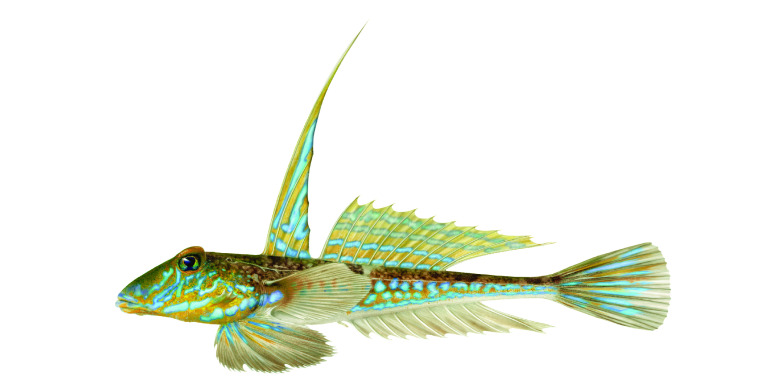
Male *Callionymus lyra*. Artwork by Karl Jilg/ArtDatabanken.

Until recently, the family Callionymidae was placed into the order Perciformes, which is often considered a ‘polyphyletic taxonomic wastebasket for families not placed in other orders’ [1]. However, recent phylogenetic analyses suggest a placement of Callionymidae within the order Syngnathiformes, which currently contains ten families with highly derived morphological characters such as the pipefish and seahorses [1]. Syngnathiformes has recently been divided into two clades, a ‘long-snouted clade’ and a ‘benthic associated clade,’ each comprising five families [2]. The ‘long-snouted clade’ (Syngnathidae, Solenostomidae, Aulostomidae, Centriscidae, and Fistulariidae) is currently represented by genomes from the Gulf Pipefish (*Syngnathus scovelli*) and the Tiger Tail Seahorse (*Hippocampus comes*) [3, 4] and additional draft assemblies of pipefish [5]. A genome of the ‘benthic associated clade’ (Callionymidae, Draconettidae, Dactylopteridae, Mullidae, and Pegasidae) has not been sequenced and analysed yet. Callionymidae comprises 196 species [6], of which the common dragonet, *Callionymus lyra* (Linnaeus, 1758) (Figure 1), is one of three *Callionymus* species inhabiting the North Sea [7]. All three species also occur in the East Atlantic, and the Mediterranean Sea [6]. They represent essential prey fish for commercially important fish species such as the cod (*Gadus morhua*) [8]. The males of the North Sea dragonet species (*C. lyra, C. maculatus, C. reticulatus*) show strong morphological differentiation in the form of species-specific colouration and size relations. The much less conspicuous females can be distinguished morphologically, with rather high inaccuracy, by the presence or absence of their preopercular, basal spine and by various percentual length ratios. The great resemblance among the different species’ females, together with the fact that all three species can be found in sympatry, suggests there is the possibility of hybridization among them.

Here, we present the chromosome-level genome of the common dragonet, representing the first genome of the ‘benthic associated’ Syngnathiformes clade as a reference for future population genomic, phylogenomic, and comparative genomic analyses. The chromosome-level genome assembly was generated as part of a six-week university master’s course. For a detailed description and outline of the course, see Prost *et al.* [9].

## Sampling, DNA extraction, and sequencing

We sampled two *Callionymus lyra* (NCBI: txid34785; Fishbase ID:23) individuals (one of each sex) during a yearly monitoring expedition to the Dogger Bank in the North Sea (Female: 54° 59.189*′* N 1° 37.586*′* E; Male: 54° 48.271*′* N 1° 25.077*′* E) with the permission of the Maritime Policy Unit of the UK Foreign and Commonwealth Office in 2019. The samples were initially frozen at −20 °C on the ship and later stored at −80 °C until further processing. The study was conducted in compliance with the ‘Nagoya Protocol on Access to Genetic Resources and the Fair and Equitable Sharing of Benefits Arising from Their Utilization’.

We extracted high molecular weight genomic DNA (hmwDNA) from muscle tissue of the female individual following the protocol by Mayjonade *et al.* [10]. Quantity and quality of the DNA was evaluated using the Genomic DNA ScreenTape on the Agilent 2200 TapeStation system (Agilent Technologies). Library preparation for long-read sequencing followed the associated protocols for Oxford Nanopore Technologies (ONT, Oxford, UK) Rapid Sequencing Kit (SQK-RAD004). A total of seven sequencing runs were performed using individual flow cells (FLO-MIN106 v.9.41) on a ONT MinION v.Mk1B.

Additionally, we sent tissue samples to BGI Genomics (Shenzhen, China) to generate additional sequencing data. A 100 bp paired-end short-read genomic DNA sequencing library was prepared from the muscle tissue of the female individual. This library was later used for genome assembly polishing. Moreover, a 100 bp paired-end RNAseq library was prepared for pooled RNA isolates derived from kidney, liver, gill, gonad, and brain tissues of the male individual. Both libraries were sequenced on BGI’s DNBseq platform (BGISEQ-500/DNBSEQ-G50 sequencing) [11]. We received a total of 159,925,221 read pairs (∼32 Gbp) of pre-filtered genomic DNA sequencing data and 61,496,990 read-pairs (∼12.3 Gbp) of pre-filtered RNAseq data.

Furthermore, we prepared a Hi-C library using the Dovetail™ Hi-C Kit (Dovetail Genomics, Santa Cruz, California, USA) from muscle tissue of the female and sent the library to Novogene Co., Ltd. (Beijing, China) for sequencing on an Illumina NovaSeq 6000. Sequencing yielded a total of 104,668,356 pre-filtered 150 bp paired-end read pairs or 31.4 Gbp of sequencing data. This data was used for proximity-ligation scaffolding of the assembly.

## Genome size estimation

We estimated the genome size for *C. lyra* using both k-mer frequencies and flow cytometry. The k-mer frequency for *K* = 21 was calculated from the short-read DNBseq data and summarized as histograms with jellyfish v.2.2.10 (RRID:SCR_005491) [12]. Plotting the histograms and calculating the genome size and heterozygosity with GenomeScope v.1.0 (RRID:SCR_017014) [13] resulted in a genome size estimate of approximately 562 Mbp. For the genome size estimation using flow cytometry, frozen muscle tissue was finely chopped with a razor blade in 200 μl LeukoSure Lyse Reagent (Beckman Coulter Inc., Fullerton, CA, USA). Large debris was removed by filtering through a 40 μm Nylon cell strainer and an RNAse treatment was performed with a final concentration of 0.3 mg/ml. Simultaneously, we stained the DNA in the nuclei with propidium iodide (PI) at a final concentration of 0.025 mg/ml and incubated the solution for 30 min at room temperature, protected from light exposure. Fluorescence intensities of the nuclei were recorded on the CytoFLEX Flow Cytometer (Beckman Coulter Inc., Fullerton, CA, USA). The domestic cricket (*Acheta domesticus,* C-value: 2.0 pg) was used as a reference to determine the genome size of *C. lyra*. For a more precise estimate we analysed five independent technical replicates resulting in an average C-value of 0.66 pg, which corresponds to a haploid genome size of approximately 645 Mbp.

## Genome assembly and polishing

Nanopore raw signal data (fast5) of the seven sequencing runs were base-called with Guppy v.3.2.4 (ONT) using the high accuracy setting. All individual sequencing runs were examined and compared with NanoComp v.1.0.0 [15] (Figure in GigaDB [14], Table 1).

**Table 1:** Read output and quality of the seven different MinION sequencing runs and the final concatenated dataset.

	Run 1	Run 2	Run 3	Run 4	Run 5	Run 6	Run 7	Total
Mean read length	1,153	1,528	2,562	2,542	1,913	1,334	1,211	1,904
Mean read quality:	8.7	9.3	10.1	10.6	10.6	9.4	9.9	10.2
Number of reads:	49,659	114,845	2,465,768	2,360,176	6,506,852	2,149,172	2,714,852	16,361,324
Read length N50:	3,425	3,628	5,469	5,257	3,485	2,880	2,179	3,931
Total bases:	57,260,945	175,534,110	6,316,560,588	5,998,757,646	12,447,462,241	2,865,915,086	3,287,474,510	31,148,965,126

The final dataset, after concatenation of all read-files, was further examined with NanoPlot v.1.0.0 (Table 1) [15]. Concatenation of all read-files resulted in a total dataset of 31 Gbp or approximately 55-fold coverage as the basis for the genome assembly.

We assembled the genome of *C. lyra* with wtdbg2 v.2.2 (RRID:SCR_017225) [16] using the default parameters for ONT reads. The resulting assembly was subjected to a three-step polishing approach. First, a single iteration of racon v.1.4.3 (RRID:SCR_017642) [17] corrected for errors typical of the MinION platform: homopolymers and repeat errors. Next, we used one iteration of medaka v.0.11.5 [18] on the racon-polished assembly. According to the developers medaka is most effective after a polishing run with racon. Following polishing with the long-read data, we used three iterations of pilon v.1.23 (RRID:SCR_014731) [19] to correct for random errors and single-base errors with the high-quality short-read data.

## Assembly QC and scaffolding

**Figure 2.**
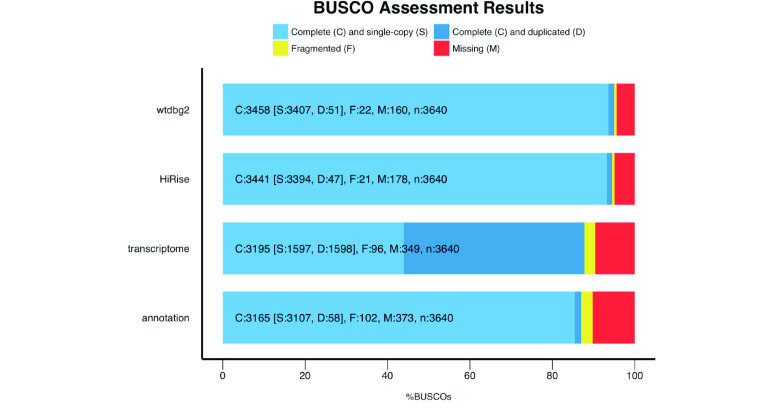
Gene completeness analysis of the long-read based contig assembly (wtdbg2), the Hi-C scaffolded assembly (HiRise), the transcriptome of *Callionymus lyra*, and the annotation. The high percentage of duplicated BUSCOs in the transcriptome is attributed to protein isoforms.

We calculated assembly continuity statistics using QUAST v.5.0.2 (RRID:SCR_001228) [20] and performed a gene set completeness analysis using BUSCO v.4.0.6 (RRID:SCR_015008) [21] with the provided database for Actinopterygii orthologous genes (actinopterygii_odb10). The final polished assembly had 1,782 contigs and a total length of 569 Mbp, which is marginally larger than the k-mer based estimate of 562 Mbp and 84 Mbp shorter than the flow cytometry estimate. This is expected, because very repetitive regions are usually missing or collapsed in a genome assembly, which could explain the shorter assembly length compared to the flow cytometry size estimate. The assembly shows a high continuity with long contigs of up to 10.7 Mbp and a contig N50 of >2.2 Mbp (Table 1). The genome assembly completeness analysis identified 95.0% complete BUSCO genes (93.6% complete, single copy) and only 4.4% missing BUSCOs, which suggests that the assembly contains most of the coding regions of the genome (Figure 2, Table 2).

**Table 2:** BUSCO results of the long-read based contig assembly (wtdbg2), Hi-C scaffolded assembly (HiRise), the transcriptome, and the annotation of the *Callionymus lyra* assembly.

	wtdbg2	HiRise^∗^	Transcriptome	Annotation
Complete BUSCOs	3458 (95.0%)	3441 (94.5%)	3195 (87.8%)	3165 (87.0%)
Complete and single-copy BUSCOs	3407 (93.6%)	3394 (93.2%)	1597 (43.9%)	3107 (85.4%)
Complete and duplicated BUSCOs	51 (1.4%)	47 (1.3%)	1598 (43.9%)	58 (1.6%)
Fragmented BUSCOs	22 (0.6%)	21 (0.6%)	96 (2.6%)	102 (2.8%)
Missing BUSCOs	160 (4.4%)	178 (4.9%)	349 (9.6%)	373 (10.2%)
Total BUSCO groups searched	3640	3640	3640	3640

^∗^Final assembly after removing contaminated scaffolds and scaffolds <200 bp.

**Figure 3.**
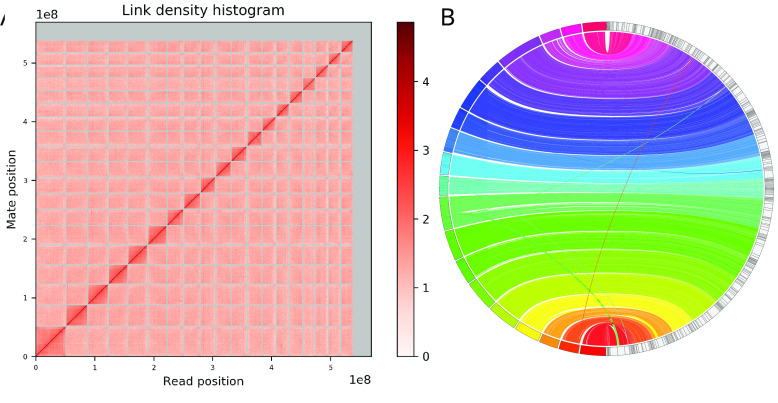
(A) Hi-C contact map of the 19 chromosome-length scaffolds, and additional unplaced scaffolds. (B) Whole genome synteny between the polished contig assembly from wtdbg2 (on the right) and the final Hi-C scaffolded chromosome-level assembly (on the left). Crossing lines indicate assembly artifacts corrected during scaffolding.

To achieve chromosome-length scaffolds, we used the long-read based assembly and the generated Hi-C data as input for the HiRise scaffolding pipeline [22] as part of the Dovetail Genomics’ scaffolding service. HiRise made 538 joins and 10 breaks resulting in a scaffolded assembly with a total of 1,254 scaffolds and a scaffold N50 of 26.7 Mbp. Over 94.5% (538 Mbp) of the total assembly length was scaffolded into 19 chromosome-length scaffolds (Figure 3A). The number of chromosome-length scaffolds is consistent with the haploid number of chromosomes derived from karyotypes of females of two Callionymidae species (*C. beniteguri* and *Repomucenus ornatipinnis*) [23]. Therefore, the number of chromosomes appears to be relatively conserved within *Callionymidae* and it is likely that *C. lyra* follows the same chromosomal sex determination system as *C. beniteguri* and *R. ornatipinnis* (♀: X_1_X_2_–X_1_X_2_ (2*n* = 38); ♂: X_1_X_2_–Y (2*n* = 37)) [23].

**Figure 4.**
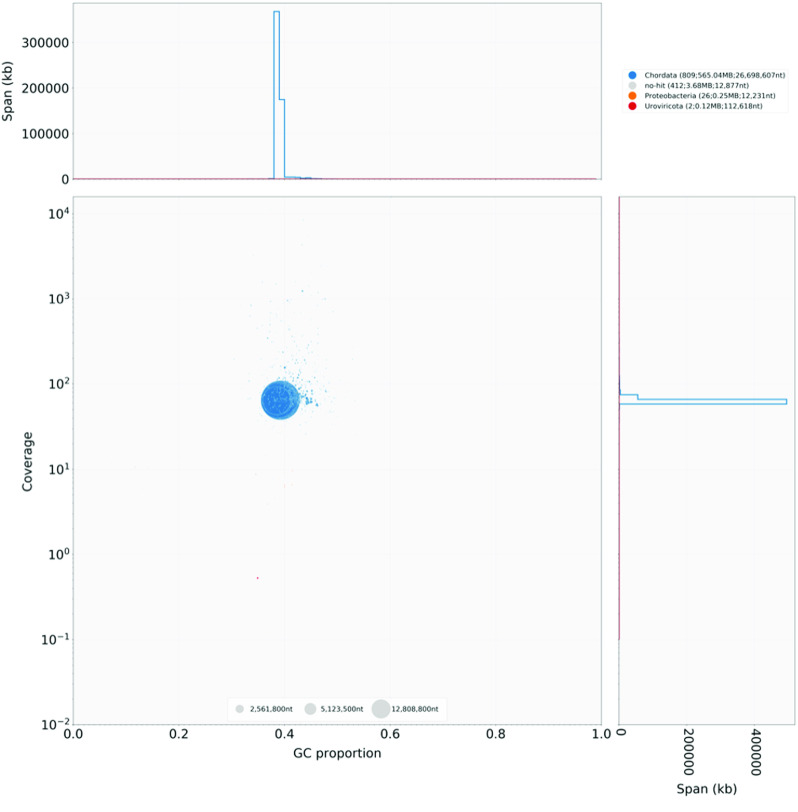
Blobtools plot showing the taxonomic assignments (blue colour for Chordata, gray for ‘no hits’, orange for Proteobacteria, and red for Uroviricota) of the different scaffolds, and scaffold-wide coverage and GC contents. The scaffolds were blasted against the NCBI nucleotide database. Scaffolds with assignments to Proteobacteria or Uroviricota were removed from the final assembly.

For a final assembly quality control, we mapped the raw nanopore reads with minimap2 v.2.17-r941 [24] and the DNBSeq data with bwa-mem v.0.7.17-r1194-dirty (RRID:SCR_010910) [25] onto the final assembly with a high mapping rate of 94.8% and 98.62%, respectively. We further checked the assembly for contamination with BlobTools v.1.1.1 (RRID:SCR_017618) [26]. This analysis identified minor contamination from Proteobacteria (26 short contigs, in total 0.25 Mbp) and Uroviricota (2 contigs, in total 0.12 Mbp) (Figure 4). No contamination was found in the 19 chromosome-length scaffolds. Subsequently, we removed all contaminations and contigs with a length of <200 bp from the final assembly (for final statistics see Table 3). In addition, we screened for mitochondrial sequence contamination with BLASTN v.2.9.0+ (RRID:SCR_001598) [27] using the available mitochondrial genome sequence of *C. lyra* (Accession No.: MN122938.1) as a reference. A single sequence of mitochondrial origin (169 bp) was identified on one scaffold. This partial mitochondrial sequence could either be an assembly artifact or nuclear mitochondrial DNA (numt).

A synteny plot comparing the polished wtdbg2 contig assembly with the final chromosome-level assembly, generated with JupiterPlot v.1.0 [28], found overall strong agreements with only few differences (Figure 3B). These likely constitute assembly errors in the contig assembly that were fixed by HiRise during scaffolding. A BUSCO analysis of the final assembly found slightly less complete BUSCO genes compared to the wdtbg2 contig assembly (94.5% vs. 95.0%) (Figure 2, Table 2).

## Transcriptome assembly and quality

In addition to the genome, we assembled the transcriptome of *C. lyra* for subsequent use in the genome annotation using Trinity v.2.9.0 (RRID:SCR_013048) [29, 30] based on the 12.3 Gbp multi-tissue RNAseq data. The resulting transcriptome assembly has a total length of 255.5 Mbp (Table 3). BUSCO analysis suggests a high transcriptome completeness with 87.8% of orthologous genes found in the transcriptome assembly (Figure 2, Table 2).

**Table 3:** Assembly statistics of the long-read based contig assembly (wtdbg2), Hi-C scaffolded assembly (HiRise) and the transcriptome assembly of *Callionymus lyra*.

	wtdbg2	HiRise^∗^	Transcriptome
No. of contigs	1,782	1,205	246,012
No. of contigs (>1 kbp)	1,707	1,151	66,332
L50	70	9	23,587
L75	176	14	49,697
N50	2,201,294 bp	26,698,546 bp	2,787 bp
N75	856,699 bp	22,283,913 bp	1,474 bp
Max. contig length	10,738,616 bp	51,234,906 bp	31,800 bp
Total length	569,037,589 bp	568,707,486 bp	255,540,591 bp
GC (%)	38.97	38.97	46.84
No. of gaps	0	57,348	0
No. of N’s per 100 kbp	0.0	10.08	0.0

^∗^Final assembly after removing contaminated scaffolds and scaffolds <200 bp.

## Genome annotation

### Repeat annotation

In order to annotate repeats in the assembly, we created a custom *de novo* repeat library using RepeatModeler v.1.0.11 (RRID:SCR_015027) [31] and combined this library with the *Actinopterygii* repeat database from RepBase. Repeats in the genome were then annotated using RepeatMasker open-4.0.7 (RRID:SCR_012954) [32]. Our analyses identified 27.66% of repeats in the genome, of which the majority consisted of DNA transposons (6.10%), LINE’s (5.32%) and simple repeats (3.47%). Additionally, 10.69% of unclassified repeats were identified (Table 4).

**Table 4:** Repeat content of the Hi-C scaffolded assembly^∗^.

Type of element	Number of elements	Length	Percentage of assembly
SINEs	11,019	1,338,491	0.24%
LINEs	117,005	30,245,708	5.32%
LTR elements	21,355	6,054,779	1.06%
DNA transposons	196,173	34,690,450	6.10%
Unclassified	346,500	60,808,984	10.69%
Small RNA	1,793	208,482	0.04%
Satellites	2,019	892,831	0.16%
Simple repeats	235,150	19,740,698	3.47%
Low complexity	28,668	1,821,423	0.32%
		Total:	27.66%

^∗^Final assembly after removing contaminated scaffolds and scaffolds <200 bp.

### Gene annotation

Prior to annotating genes, interspersed repeats in the genome were hard-masked and simple repeats soft-masked to increase the accuracy and efficiency of locating genes. Gene annotation was performed using MAKER2 v.2.31.10 (RRID:SCR_005309) [33]. First, evidence-based annotation was conducted using a combination of *de novo* assembled transcriptomes and homologous gene identification based on previously published proteins of the Tiger Tail Seahorse (*Hippocampus comes*) [3] and the SwissProt protein database [34]. Next, genes were *ab initio* predicted with SNAP v.2006-07-28 (RRID:SCR_002127) [35] and Augustus v.3.3 (RRID:SCR_008417) [36]. The final gene annotation resulted in 19,849 transcripts, which is slightly lower compared to the number of transcripts in the Gulf Pipefish genome (20,841) and Tiger Tail Seahorse genome (22,941) [3, 4]. Of all identified gene models, 96% had an AED score of ≤ 0.5 (AED score distributions in GigaDB [14]), indicating a high quality of the annotated gene models [37]. In addition, BUSCO analysis identified 87.0% complete BUSCOs, which suggest a high completeness of the annotation (Figure 2, Table 2).

## Conclusion

Here we report the first genome assembly of the ‘benthic associated’ Syngnathiformes clade, the sister group to the ‘long-snouted clade’ (e.g., seahorses and pipefish). The annotated genome of *Callionymus lyra*, with its high continuity (chromosome-level), provides an essential reference to study speciation and potential hybridization in Callionymidae and is an important resource for phylogenomic analyses among syngnathiform fish.

## Data Availability

All raw data generated in this study including Nanopore long-reads, DNBSeq short-reads, Hi-C reads, and RNASeq data, and the chromosome-level assembly are accessible at GenBank under BioProject PRJNA634838. Annotation, results files and other data are available in the GigaDB repository [14].

## List of abbreviations

BLASTN: Basic Local Alignment Search Tool (for nucleotides); bp: base pairs; BUSCO: Benchmarking Universal Single-Copy Orthologs; DNBSeq: DNA NanoBall sequencing; Gbp: Gigabase pairs; hmwDNA: high molecular weight DNA; kbp: kilobase pairs; Mbp: megabase pairs; numt: nuclear mitochondrial DNA; ONT: Oxford Nanopore Technologies; pg: picogram; PI: propidium iodide; RNAseq: RNA sequencing.
